# A shoot derived long distance iron signal may act upstream of the IMA peptides in the regulation of Fe deficiency responses in *Arabidopsis thaliana* roots

**DOI:** 10.3389/fpls.2022.971773

**Published:** 2022-08-29

**Authors:** María José García, Macarena Angulo, Francisco Javier Romera, Carlos Lucena, Rafael Pérez-Vicente

**Affiliations:** ^1^Department of Agronomy (DAUCO-María de Maeztu Unit of Excellence), Campus de Excelencia Internacional Agroalimentario, Universidad de Córdoba, Córdoba, Spain; ^2^Department of Botany, Ecology and Plant Physiology, Campus de Excelencia Internacional Agroalimentario, Universidad de Córdoba, Córdoba, Spain

**Keywords:** dicotyledonous, ethylene, Fe deficiency responses, IMA peptides, Long Distance Iron Signal (LODIS), Strategy I plants

## Abstract

When plants suffer from Fe deficiency, they develop morphological and physiological responses, mainly in their roots, aimed to facilitate Fe mobilization and uptake. Once Fe has been acquired in sufficient quantity, the responses need to be switched off to avoid Fe toxicity and to conserve energy. Several hormones and signaling molecules, such as ethylene, auxin and nitric oxide, have been involved in the activation of Fe deficiency responses in Strategy I plants. These hormones and signaling molecules have almost no effect when applied to plants grown under Fe-sufficient conditions, which suggests the existence of a repressive signal related to the internal Fe content. The nature of this repressive signal is not known yet many experimental results suggest that is not related to the whole root Fe content but to some kind of Fe compound moving from leaves to roots through the phloem. After that, this signal has been named LOng-Distance Iron Signal (LODIS). Very recently, a novel family of small peptides, “IRON MAN” (IMA), has been identified as key components of the induction of Fe deficiency responses. However, the relationship between LODIS and IMA peptides is not known. The main objective of this work has been to clarify the relationship between both signals. For this, we have used Arabidopsis wild type (WT) Columbia and two of its mutants, *opt3* and *frd3*, affected, either directly or indirectly, in the transport of Fe (LODIS) through the phloem. Both mutants present constitutive activation of Fe acquisition genes when grown in a Fe-sufficient medium despite the high accumulation of Fe in their roots. Arabidopsis WT Columbia plants and both mutants were treated with foliar application of Fe, and later on the expression of IMA and Fe acquisition genes was analyzed. The results obtained suggest that LODIS may act upstream of IMA peptides in the regulation of Fe deficiency responses in roots. The possible regulation of IMA peptides by ethylene has also been studied. Results obtained with ethylene precursors and inhibitors, and occurrence of ethylene-responsive cis-acting elements in the promoters of IMA genes, suggest that IMA peptides could also be regulated by ethylene.

## Introduction

Iron (Fe) participates in many important processes of plants, such as photosynthesis, respiration and nitrogen metabolism (Marschner, 2012). Iron is abundant in most soils, but its availability for plants is low, especially in calcareous soils, where the incidence of Fe chlorosis is frequent (Briat et al., 2015). Since calcareous soils represent more than 30% of arable soils, Fe chlorosis is one of the most important deficiencies caused by a micronutrient worldwide. Under Fe-deficient conditions, Strategy I plants (all plants but grasses) induce morphological and physiological changes in their roots, known as Fe deficiency responses (Kobayashi and Nishizawa, 2012; Brumbarova et al., 2015; Lucena et al., 2015), which favor Fe mobilization and acquisition. Among the main physiological responses are: an enhanced ferric reductase activity (FRA; determined by *FRO* genes), an enhanced Fe(II) uptake capacity (determined by *IRT* genes) and the acidification of the rhizosphere (Kobayashi and Nishizawa, 2012; Brumbarova et al., 2015). Among the morphological responses are: development of root hairs, of cluster roots and of transfer cells, all of them aimed to increase the surface of contact with soil (Lucena et al., 2015; Romera et al., 2017).

In the last years, the knowledge of the regulatory network controlling Fe deficiency responses and Fe acquisition by plants has increased considerably. In Arabidopsis (Strategy I), the master regulator of most Fe acquisition genes is FIT (bHLH29), a basic-helix–loop–helix transcription factor (TF), homologous of the tomato FER (Ling et al., 2002; Colangelo and Guerinot, 2004). For regulating key Fe acquisition genes, like *FRO2* and *IRT1*, FIT interacts with another bHLH TFs of the Ib subgroup, such as bHLH38, bHLH39, bHLH100 and bHLH101. All of them are induced in roots in response to Fe deficiency and have redundant function (Yuan et al., 2008; Wang et al., 2013; Brumbarova et al., 2015). The expression of *FIT*, *bHLH38*, *bHLH39*, *bHLH100* and *bHLH101* is induced by homo- and heterodimers formed by IVc subgroup bHLH TFs: bHLH105 (ILR3) and its closest homologs bHLH34, bHLH104 and bHLH115 (Zhang et al., 2015; Li et al., 2016; Liang et al., 2017; Gao et al., 2019). Upstream of the IVc subgroup there are other bHLH TFs, like bHLH121 (URI), and the BRUTUS (BTS) and BTS-LIKE (BTSL) proteins (Zhang et al., 2015; Hindt et al., 2017; Kim et al., 2019; Rodríguez-Celma et al., 2019; Gao et al., 2020). BTS and BTSL proteins are E3 ligases which act as potential Fe sensors that interact with IVc bHLH TFs and FIT, targeting them for proteasomal degradation (Zhang et al., 2015; Liang et al., 2017; Rodríguez-Celma et al., 2019). Since FIT and the IVc bHLH TFs act as positive regulators of Fe deficiency responses, BTS and BTSL proteins would act as negative regulators (Zhang et al., 2015; Hindt et al., 2017; Rodríguez-Celma et al., 2019; Li et al., 2021).

Several hormones and signaling substances, whose production increases in Fe-deficient roots, have been implicated in the activation of Fe deficiency responses. Among them, ethylene plays a prominent role by up-regulating up to 19 Fe deficiency induced genes, including main regulatory genes, like *FIT*, *bHLH38* and *bHLH39*, as well as essential genes for Fe acquisition (*FRO2* and *IRT1*) and distribution (*FRD3, NAS1* and *NAS2*; Lucena et al., 2006, 2015; Waters et al., 2007; García et al., 2010, 2011, 2018; Lingam et al., 2011; Romera et al., 2011, 2017; Yang et al., 2014; Li and Lan, 2017). Ethylene has also been involved in the regulation of morphological responses to Fe deficiency (Romera et al., 2011, 2017; Lucena et al., 2015) and in restricting the suberization of the endodermis under Fe deficiency (Barberon et al., 2016). Ethylene, auxin and other signaling substances, like NO (nitric oxide), greatly activate the expression of Fe acquisition genes in plants grown with low levels of Fe but weakly in those grown with high levels of Fe (Lucena et al., 2006; Graziano and Lamattina, 2007; Chen et al., 2010; Bacaicoa et al., 2011; García et al., 2011). This suggests the existence of a repressive signal related to the internal Fe content. The nature of this repressive signal is not yet known but many experimental results suggest that is not related to the whole root Fe content but to some kind of Fe compound moving from leaves to roots through the phloem. After that, this signal has been named LOng-Distance Iron Signal (LODIS; García et al., 2018). Although the nature of LODIS is not known, its consequences have already been investigated by comparing LODIS-deficient mutants, such as Arabidopsis *frd3* and *opt3*, with the wild type (WT) cultivar Columbia, and by comparing plants treated or not with foliar application of Fe.

The Arabidopsis *frd3* and *opt3* mutants present constitutive activation of Fe acquisition genes (Rogers and Guerinot, 2002; Stacey et al., 2008; García et al., 2013). The *frd3* mutant is affected in xylem Fe transport, being chlorotic even when grown in a Fe-sufficient medium (Durrett et al., 2007). However, it becomes green, and its Fe acquisition genes are down-regulated, when treated with Fe in the leaves, which indicates that LODIS can move from leaves to roots through its phloem (Lucena et al., 2006). However, the foliar application of Fe does not down-regulate Fe acquisition genes in *opt3* plants, indicating that the *opt3* mutation impairs LODIS translocation from leaves to roots (García et al., 2013, 2018). The Arabidopsis mutant *opt3* is impaired in the loading of Fe and Cu into the phloem (Stacey et al., 2008; García et al., 2013, 2018; Chia et al., 2021). *OPT3*, whose expression, mainly in shoots, is enhanced under Fe or Cu deficiency, belongs to the oligopeptide transporter (OPT) family (Stacey et al., 2008; Chia et al., 2021). The nature of the substrate transported through OPT3 is not yet clear. Some authors, such as Zhai et al. (2014) and Chia et al. (2021), have found that OPT3 can transport Fe^2+^ and Cu^2+^ ions when expressed in *Xenopus* oocytes. However, other authors, like Mendoza-Cózatl et al. (2014), have found that OPT3 is unable to rescue the *fet3fet4* strain of yeast, impaired in Fe uptake. In any case, even if OPT3 transports Fe ions, these ones should be chelated in the phloem sap to avoid their precipitation (Gutiérrez-Carbonell et al., 2015). GSH-derived compounds, proteins and peptides are among the proposed chelating agents (Krüger et al., 2002; Ramírez et al., 2011; Darbani et al., 2013; García et al., 2013).

Recently, a family of small peptides named IRON MAN/FE-UPTAKE-INDUCING PEPTIDE (IMA/FEP) has been shown to play a key role in the regulatory network of Fe acquisition and cellular homeostasis in plants (Grillet et al., 2018; Hirayama et al., 2018; Gautam et al., 2021; Kobayashi et al., 2021; Li et al., 2021). The *Arabidopsis* genome harbours eight *IMA* genes, all of them responsive to Fe supply. *IMA1*, *IMA2* and *IMA3* are highly expressed in both leaves and roots of Fe-deficient plants (Grillet et al., 2018). *IMA1*, *IMA2* and *IMA3* expression is under the transcriptional control of bHLH TFs, like bHLH105, bHLH115 or bHLH121 (see above; Gao et al., 2020; Li et al., 2021). Moreover, IMA peptides are ubiquitinated by BTS or BTSL proteins and degraded *via* the proteasome (Li et al., 2021; Lichtblau et al., 2022). These authors propose that IMA peptides could impair the interaction of BTS and BTSL proteins with IVc bHLH TFs, thus diminishing their proteasomal degradation and, consequently, favoring the activation of Fe deficiency responses in Arabidopsis roots (Li et al., 2021; Lichtblau et al., 2022).

IMA peptides function is redundant: *ima8x* plants are very small, chlorotic and died few days when grown under Fe sufficient conditions; however, the overexpression of *IMA1* in *ima8x* plants restored the growth, chlorophyll content and the ferric reductase activity (Grillet et al., 2018). The results obtained by RNAseq experiments carried out in *IMA* overexpressing lines (IMA1Ox) showed that most of the main regulators of Fe deficiency responses, such as *bHLH38, bHLH39, bHLH100* and *bHLH101*, and genes involved in Fe uptake (*FRO2* and *IRT1*) or distribution (*NAS1*, *NAS2* and *FRD3*) are strongly induced in roots of Fe-sufficient IMA1Ox plants. Genes encoding important proteins for Fe storage, such as the ferritins *FER1* and *FER3*, and the vacuolar Fe transporters *VTL1*, *VTL2* and *VTL5*, are also upregulated in IMA1Ox plants (Grillet et al., 2018). The ectopic expression of *IMA1* and *IMA2* also induces the biosynthesis and secretion of fraxetin, a coumarin, through the induction of the *MYB72* TF and the *S8H* gene, encoding the Scopoletin 8-Hydroxylase enzyme (Gautam et al., 2021). Taken together, all these results clearly support a role for IMA peptides as activators of Fe deficiency responses in Arabidopsis roots.

Besides Arabidopsis, two *IMA*/*FEP* genes have been described in rice (Kobayashi et al., 2021). The expression of *OsIMA1* and *OsIMA2* is strongly induced under Fe deficiency, and the overexpression of *OsIMA1* or *OsIMA2* in rice confers tolerance to Fe deficiency and accumulation of Fe in leaves and seeds. The *OsIMA*-overexpressing lines exhibit enhanced expression of most of the known Fe deficiency-inducible genes involved in Fe uptake and translocation (Kobayashi et al., 2021). As occurs in Arabidopsis, *OsIMA1* and *OsIMA2* expression is also regulated by bHLH TFs, such as OsbHLH058 and OsbHLH059, and IMA proteins are degraded upon interaction with HRZ proteins (BTS homologous; Kobayashi et al., 2021; Peng et al., 2022).

IMA peptides have been described few years ago and, although some details about their regulation are known, there is still a long way to go. The relationship between IMA peptides and LODIS, as well as the relationship between IMA peptides and other signals involved in the regulation of Fe deficiency responses, such as ethylene, has not been studied yet. To clarify their possible interactions is the main objective of this work.

## Materials and methods

### Plant materials, growth conditions and treatments

*Arabidopsis thaliana* seeds from (L.) Heynh ecotype ‘Columbia’, and from its LODIS-deficient mutants *opt3-2* and *frd3-3*, were germinated and grown under controlled conditions as previously described (Lucena et al., 2006; García et al., 2018). The *opt3-2* mutant is impaired in the loading of Fe^2+^ ions into the phloem (Stacey et al., 2008; Zhai et al., 2014; Chia et al., 2021). The *frd3-3* mutant is impaired in xylem Fe transport (Rogers and Guerinot, 2002) but, as a consequence, less Fe gets to leaves to enter the phloem (Lucena et al., 2006; García et al., 2018). Briefly, seeds were germinated in black peat and, when appropriate, seedlings were transferred to individual containers (70 mL volume) with complete nutrient solution continuously aerated. The nutrient solution without Fe had the following composition: macronutrients, 2 mM Ca(NO_3_)_2_, 0.75 mM K_2_SO_4_, 0.65 mM MgSO_4_, 0.5 mM KH_2_PO_4_; and micronutrients, 50 μM KCl, 10 μM H_3_BO_3_, 1 μM MnSO_4_, 0.5 μM CuSO_4_, 0.5 μM ZnSO_4_, 0.05 μM (NH_4_)_6_Mo_7_O_24_. Fe-EDDHA was added to the nutrient solution at different concentrations (10 or 40 μM Fe-EDDHA) depending on the experiments. Plants were grown in a growth chamber at 22°C day/20°C night, with relative humidity between 50 and 70%, and an 8 h photoperiod (to postpone flowering) at a photosynthetic irradiance of 300 μMol m^−2^ s^−1^ provided by white fluorescent light (10,000 lux).

The treatments imposed were: Fe40: nutrient solution with 40 μM Fe-EDDHA; Fe40 + FeSO_4_: Fe40 treatment with FeSO_4_ application to leaves; Fe10: nutrient solution with 10 μM Fe-EDDHA; Fe10 + ACC1: Fe10 treatment with ACC addition, at 1 μM final concentration, during the last 6 or 24 h; -Fe: nutrient solution without Fe for 6, 12, 24, 48 or 72 h depending on the experiments; -Fe + Co: -Fe treatment 48 h with CoSO_4_ addition at 50, 75 or 100 μM final concentration, during the last 24 h; and-Fe + FeSO_4_: -Fe treatment during 2 days with FeSO_4_ application to leaves. For treatments with FeSO_4_, leaves were sprayed until total moistening once 24 h before harvest. FeSO_4_ was dissolved in deionized water (0.05% w/v) and Tween 20 was added as surfactant. After treatments, root ferric reductase activity (FRA) was determined as described in the next section. Finally, the roots were collected in liquid nitrogen and kept at −80°C to later analyze gene expression. Each treatment consists of six biological replications.

### Ferric reductase activity determination

Ferric reductase activity (FRA) was determined as previously described (Lucena et al., 2006). In brief, intact plants were pretreated for 30 min in plastic vessels with 50 mL of a nutrient solution without micronutrients, pH 5.5, and then placed into 20 mL of a Fe (III) reduction assay solution for 1 h. This assay solution consisted of nutrient solution without micronutrients, 100 μM Fe(III)-EDTA and 300 μM Ferrozine, pH 5.0 (adjusted with 0.1 N KOH). The environmental conditions during the measurement of Fe (III) reduction were the same as the growth conditions described above. FRA was determined spectrophotometrically by measuring the absorbance (562 nm) of the Fe(II)-Ferrozine complex and by using an extinction coefficient of 29,800 M^−1^ cm^−1^. After the reduction assay, roots were excised and weighed, and the results were expressed on a root fresh weight basis. The values represent the mean ± SE of six replicates.

### Real-time PCR analysis

PCR analysis was performed as previously described (García et al., 2018). In brief, once total RNA was extracted using the Tri Reagent solution (Molecular Research Center, Inc., Cincinnati, OH, United States), cDNA synthesis was done by using M-MLV reverse transcriptase (Promega, Madison, WI, United States) from 3 μg of DNase-treated root RNA as the template and random hexamers as the primers. The gene expression study by qRT-PCR was performed in a qRT-PCR Bio-Rad CFX connect thermal cycler and the following amplification profile: initial denaturation and polymerase activation (95°C for 3 min), amplification and quantification repeated 40 times (90°C for 10 s, 57°C for 15 s and 72°C for 30 s), and a final melting curve stage of 65 to 95°C with increment of 0.5°C for 5 s, to ensure the absence of primer dimer or non-specific amplification products. PCR reactions were set up in 20 μl of SYBR Green Bio-RAD PCR Master Mix, following the manufacturer’s instructions. Controls containing water instead of cDNA were included to check for contamination in the reaction components. Primer pairs designed by García et al. (2018) were used to amplify *FRO2*, *IRT1* and *FIT* cDNA. Standard dilution curves were performed for each primer pair to confirm appropriate efficiency of amplification (E = 100 ± 10%). *IMA1*, *IMA2* and *IMA3* genes were amplified by using the primers previously described by Hirayama et al. (2018). Constitutively expressed *SAND1* and *YLS8* genes, which do not respond to Fe supply (Han et al., 2013), were used as reference genes to normalize qRT-PCR results. The relative expression levels were calculated from the threshold cycles (Ct) values and the primer efficiencies by the Pfaffl method (Pfaffl, 2001). Each PCR analysis was conducted on three biological replicates (each biological replicate was the mixture of the roots of two plants) and each PCR reaction repeated twice.

### Occurrence of ethylene-responsive cis-acting elements in the promoters of IMA genes

The determination of ethylene-responsive cis-acting elements in the promoters of IMA genes was performed using the PLACE (Plant Cis-acting Regulatory DNA Elements) database (Higo et al., 1999).^1^ The sequences of the promoter regions of the tested genes were obtained from the EnsemblPlant database^2^ and the TAIR database.^3^

### Statistical analyses

**Figure 1:**
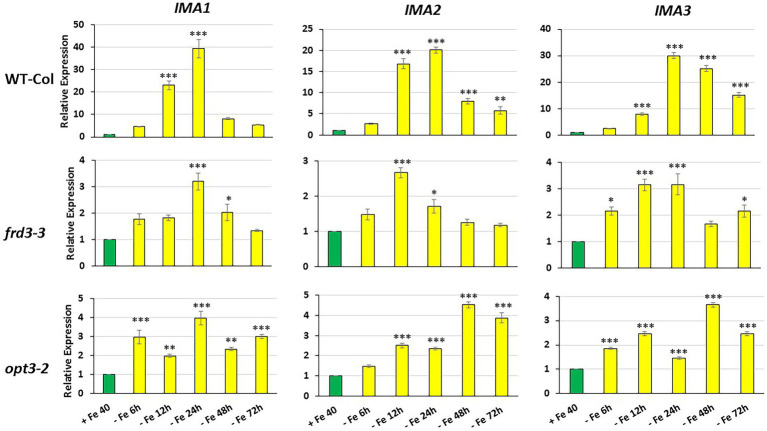
Time course of *IMA1*, *IMA2* and *IMA3* expression in roots of Arabidopsis WT Columbia and its LODIS defective mutants *frd3-3* and *opt3-2* grown under Fe-deficient conditions. Plants were grown in complete nutrient solution. When appropriate, some of them were transferred to complete nutrient solution with 40 μM Fe-EDDHA (+Fe40) or without Fe (–Fe) for 6, 12, 24, 48 or 72 h. *IMA1*, *IMA2* and *IMA3* relative expression was determined by qRT-PCR. Relative expression was calculated in relation to the +Fe40 treatment. Data represent the mean of 3 independent biological replicates ± S.E. Bars with *, ** or *** indicate significant differences (*p* < 0.05, *p* < 0.01 or *p* < 0.001, respectively) in relation to the +Fe40 treatment according to the Dunnett’s test.

**Figure 2:**
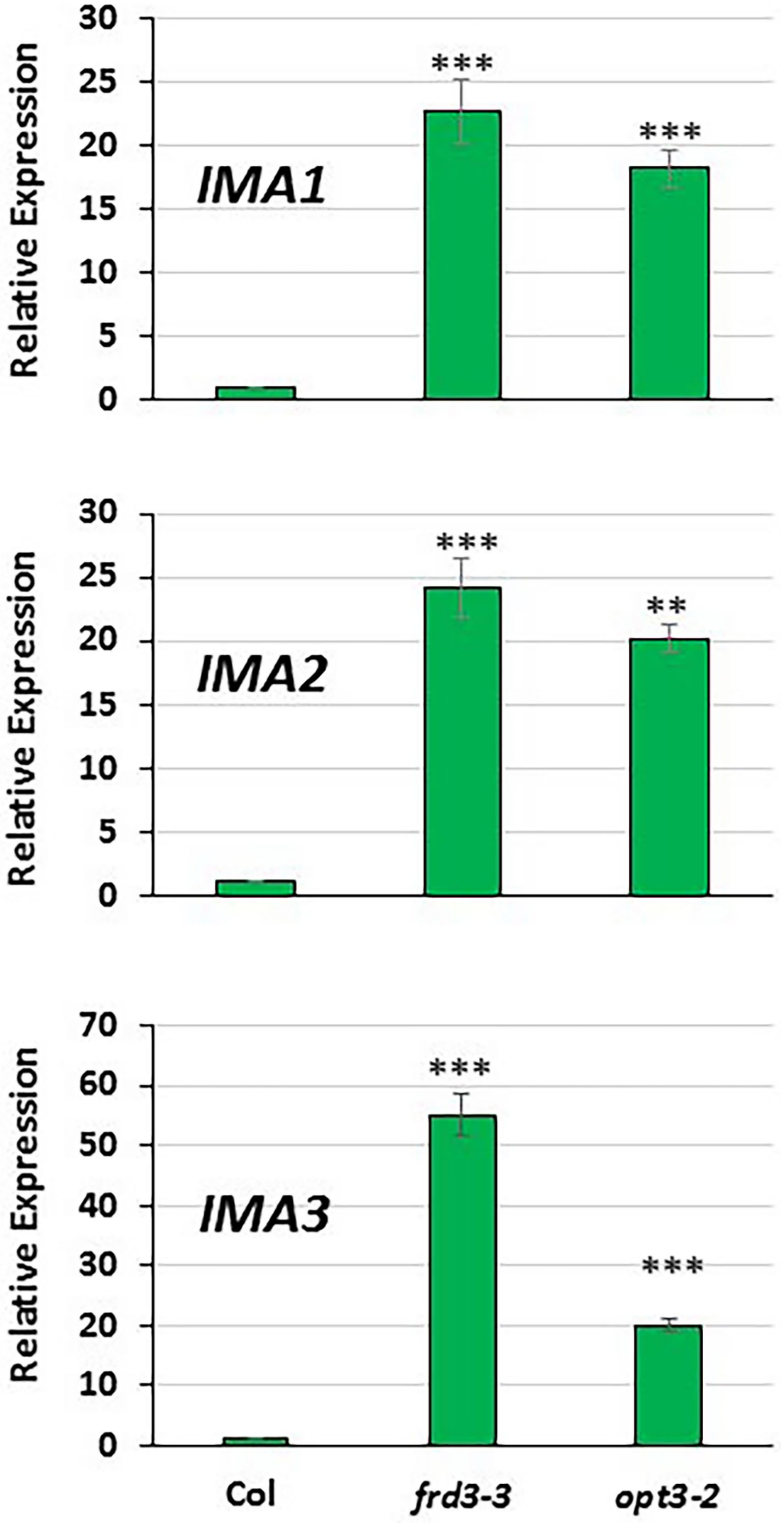
Comparison of *IMA1*, *IMA2* and *IMA3* expression in roots of Arabidopsis WT Columbia and its LODIS defective mutants *frd3-3* and *opt3-2* grown under Fe-sufficient conditions. Plants were grown in complete nutrient solution. When appropriate, were transferred to complete nutrient solution with 40 μM Fe-EDDHA (+Fe40) during 48 h. *IMA1*, *IMA2* and *IMA3* expression was determined by qRT-PCR. Relative expression was calculated in relation to the WT Columbia. Data represent the mean of 3 independent biological replicates ± S.E. Bars with ** or *** indicate significant differences (*p* < 0.01 or *p* < 0.001, respectively) in relation to the WT Colombia +Fe40 treatment according to the Dunnett’s test.

**Figure 3:**
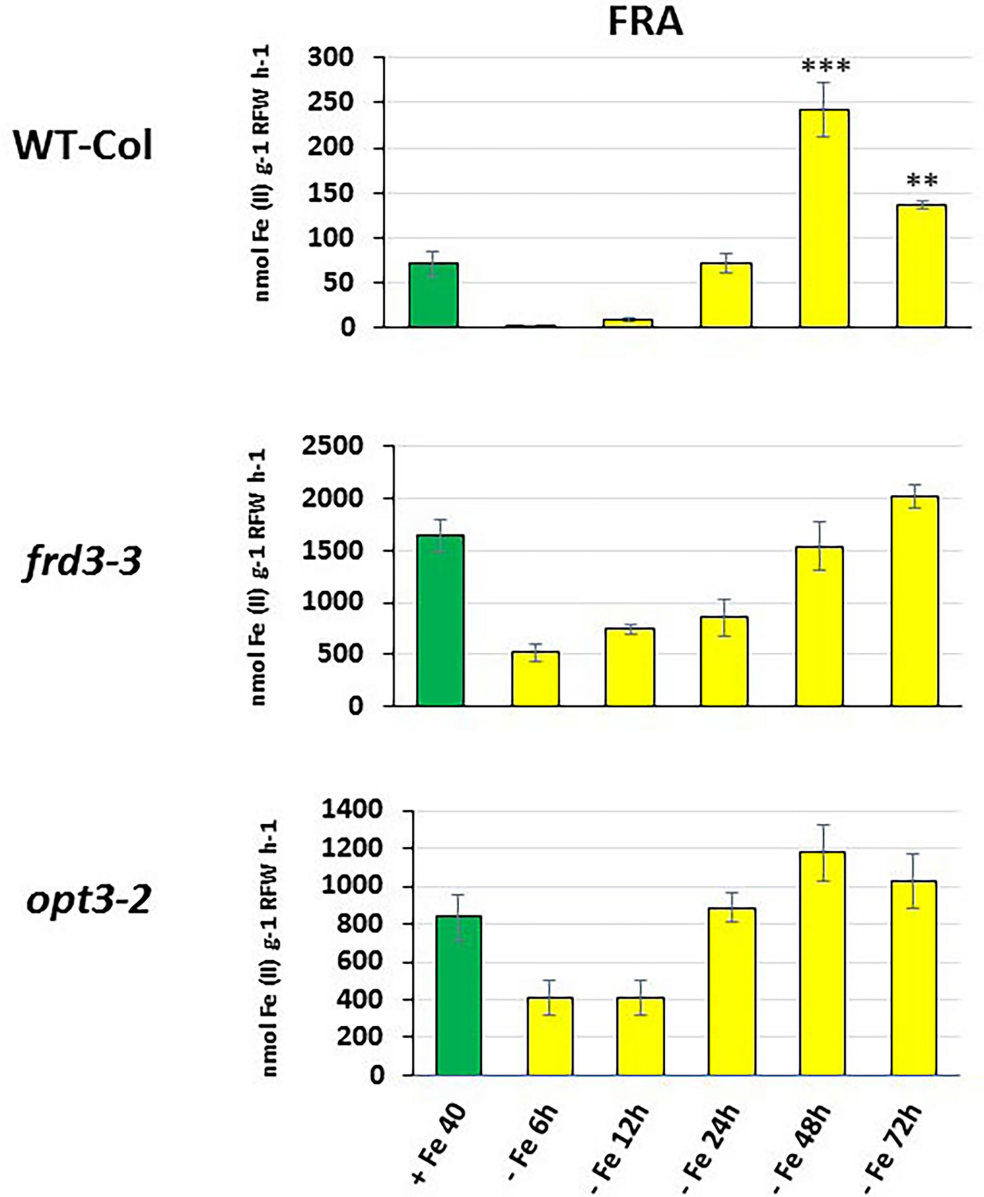
Time course of ferric reductase activity (FRA) in roots of Arabidopsis WT Columbia and its LODIS defective mutants *frd3-3* and *opt3-2* grown under Fe-deficient conditions. Treatments as in Figure 1. After treatments, FRA was determined along time. Data represent the mean of 6 replicates ± S.E. Bars with ** or *** indicate significant differences (*p* < 0.01 or *p* < 0.001, respectively) in relation to the +Fe40 treatment according to the Dunnett’s test.

**Figure 4:**
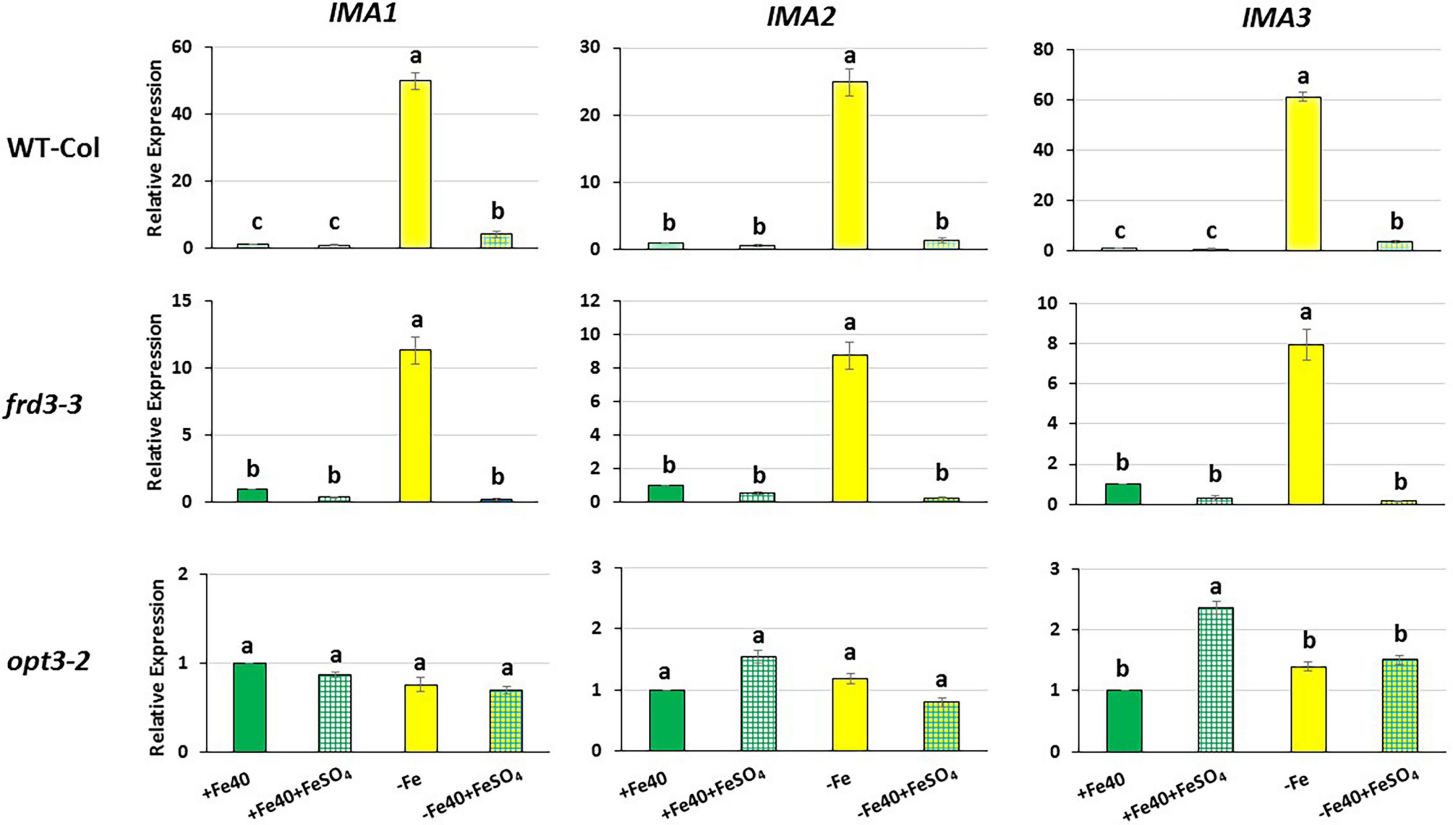
Effect of the foliar application of Fe on *IMA1*, *IMA2* and *IMA3* expression in roots of Arabidopsis WT Columbia and its LODIS defective mutants *frd3-3* and *opt3-2* grown under Fe-sufficient and Fe-deficient conditions. Plants were grown in complete nutrient solution. When appropriate, some of them were transferred to complete nutrient solution with 40 μM Fe-EDDHA (+Fe40) or without Fe (–Fe). 24 h later, half of the plants of each treatment (+Fe 40 and –Fe) were sprayed with FeSO_4_ (0.05% w/v) on their leaves (+Fe40 + FeSO_4_; –Fe + FeSO_4_). 24 h after the foliar treatments, roots were collected and *IMA1*, *IMA2* and *IMA3* expression was determined by qRT-PCR. Relative expression was calculated in relation to the +Fe40 treatment. Data represent the mean of 3 independent biological replicates ± S.E. Bars with different letters indicate significant differences (*p* < 0.05) using one-way analysis of variance (ANOVA) followed by a Tukey’s test.

**Figure 5:**
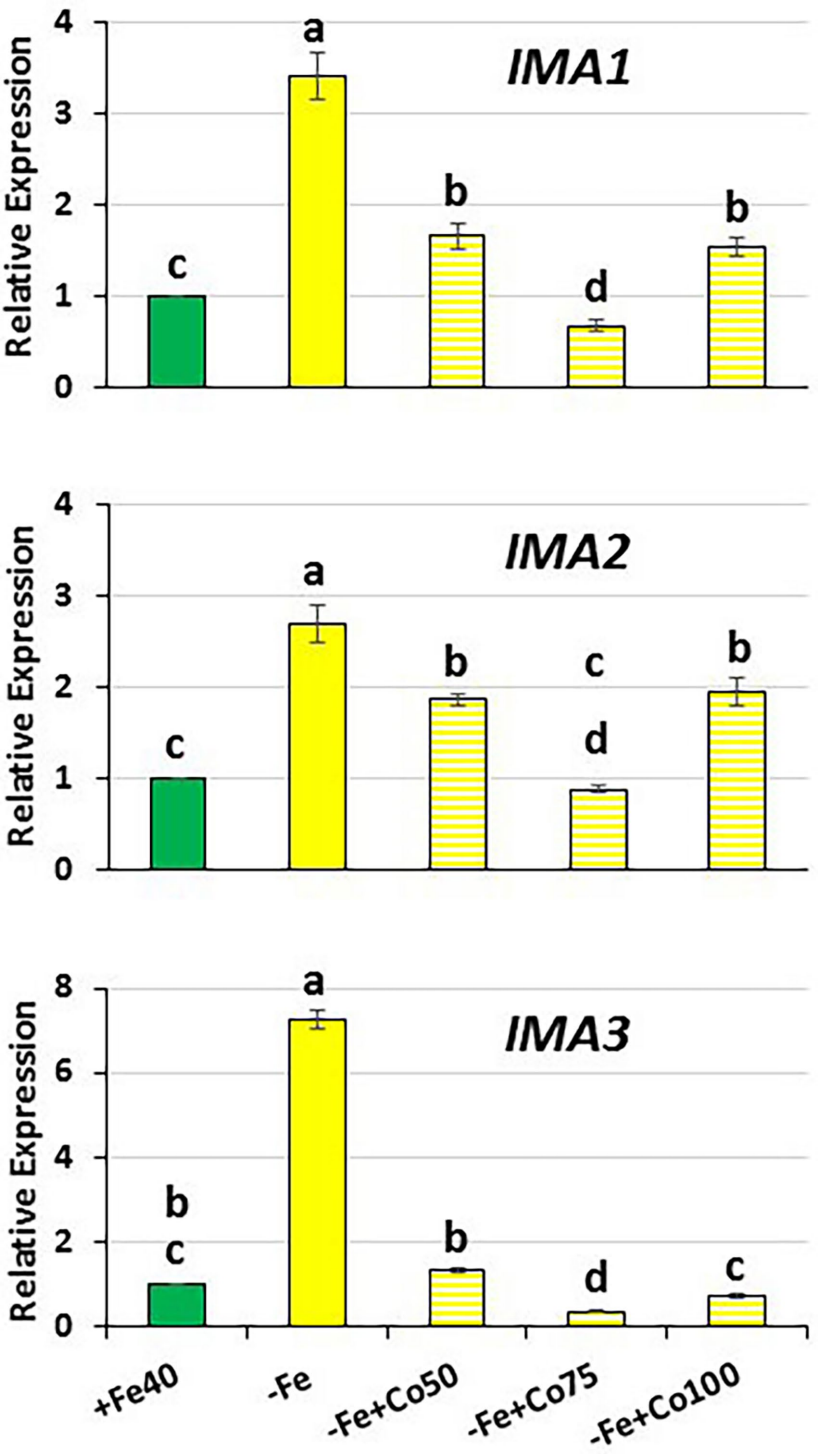
Effect of Fe deficiency and cobalt on *IMA1*, *IMA2* and *IMA3* expression in roots of Arabidopsis WT Columbia. Plants were grown in complete nutrient solution. When appropriate, some of them were transferred during 48 h to complete nutrient solution with 40 μM Fe-EDDHA (+Fe40) or without Fe (–Fe). Co, at different final concentrations (50, 75 or 100 μM), was added to the nutrient solution without Fe during the last 24 h. *IMA1*, *IMA2* and *IMA3* expression was determined by qRT-PCR. Relative expression was calculated in relation to the +Fe40 treatment. Data represent the mean of 3 independent biological replicates ± S.E. Bars with different letters indicate significant differences (*p* < 0.05) using one-way analysis of variance (ANOVA) followed by a Tukey’s test.

**Figure 6:**
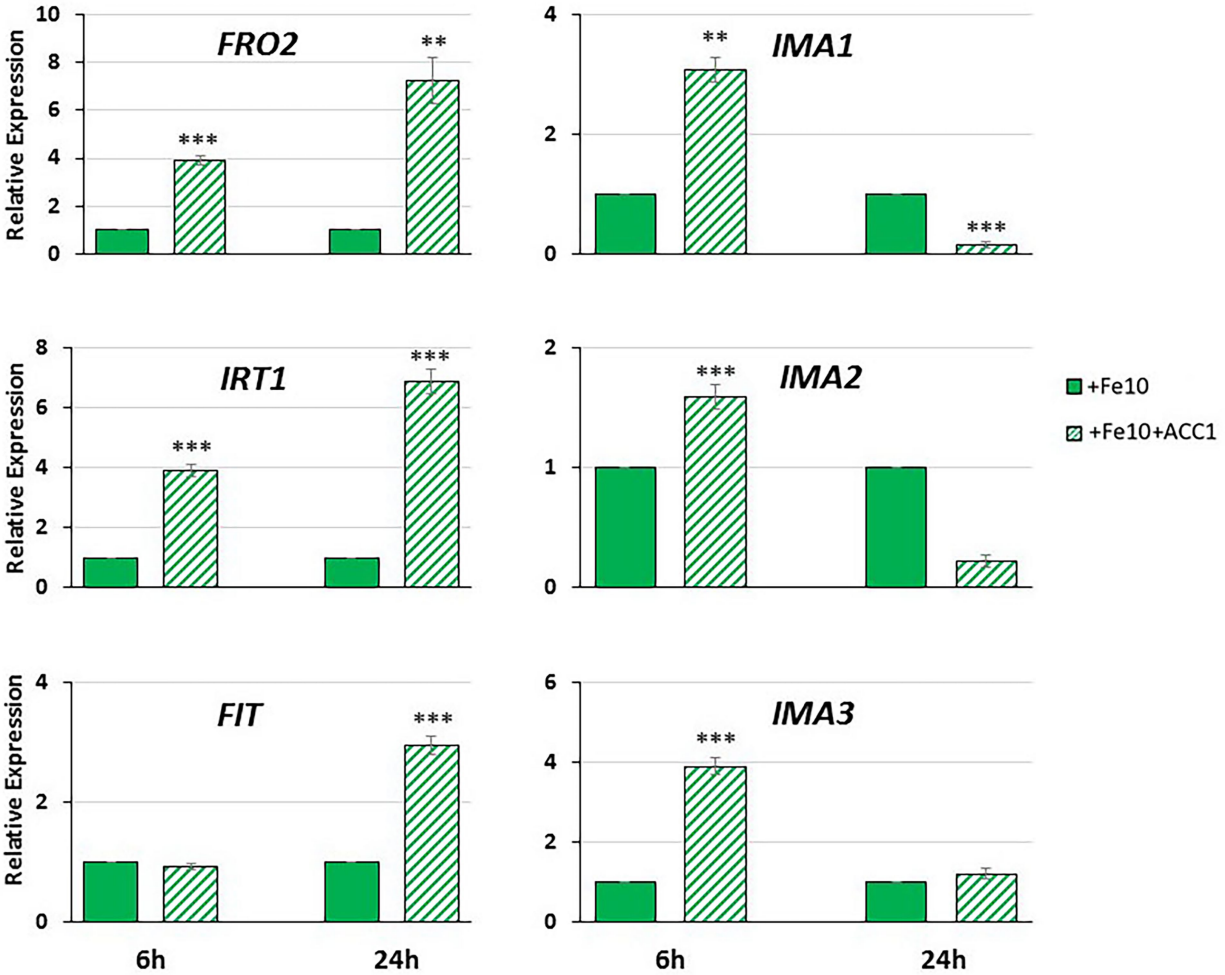
Effect of ACC treatment on the expression of the Fe acquisition genes *FRO2, IRT1* and *FIT*, and of the IMA peptide genes *IMA1*, *IMA2* and *IMA3*, in roots of Arabidopsis WT Columbia grown under Fe-sufficient conditions. Plants were grown in complete nutrient solution with 10 μM Fe-EDDHA (+Fe10). ACC at 1 μM, final concentration, was added to the nutrient solution of half of the plants during the last 6 h or 24 h. *IMA1*, *IMA2* and *IMA3* expression was determined by qRT-PCR. Relative expression was calculated in relation to the +Fe10 treatment. Data represent the mean of 3 independent biological replicates ± S.E. ** or *** indicates significant differences (*p* < 0.01 or p < 0,001, respectively) in relation to their respective control (+Fe10 6 h or + Fe10 24 h), using a Student’s test *t*.

All experiments were repeated at least twice and representative results are presented. The values of qRT-PCR represent the mean ± SE of three independent biological replicates. The values of FRA represent the mean ± SE of six replicates. Depending on the experiment, different tests were used. When comparing different treatments with a control (Figures 1–3), *, ** or *** indicate significant differences (*p* < 0.05, *p* < 0.01 or p < 0,001, respectively) using one-way analysis of variance (ANOVA) followed by a Dunnett’s test. In the foliar Fe and Co experiments (Figures 4, 5) different letters indicate significant differences (*p* < 0.05) using one-way analysis of variance (ANOVA) followed by a Tukey’s test. In ACC experiments (Figure 6) *, ** or *** indicates significant differences (*p* < 0.05, *p* < 0.01 or *p* < 0,001, respectively) in relation to their respective control (Fe10 6 h or Fe10 24 h), using a Student’s test t.

## Results

### Relationship between LODIS and IMA peptides

To look further in the possible interaction between LODIS and IMA peptides, we carried out a time course experiment to compare the relative *IMA1*, *IMA2* and *IMA3* expression in Arabidopsis WT Columbia and LODIS-deficient mutants *frd3-3* and *opt3-2* plants subjected to Fe deficiency. Complementarily, we studied the effect of the foliar application of Fe on the relative *IMA1*, *IMA2* and *IMA3* expression in the WT Columbia and the two mutants named above.

As shown in Figure 1, the relative *IMA1*, *IMA2* and *IMA3* expression greatly increased in roots of WT Columbia plants after 12 h of Fe starvation and reached their maximum expression after 24 h of the deficiency. Expression levels were up to 20–30 fold higher in Fe-deficient conditions compared to the Fe-sufficient control (Figure 1). In the mutants *frd3-3* and *opt3-2*, the differences observed in *IMA* expression under Fe-deficient conditions, relative to their control with Fe, were much lower (3–4 fold higher) than the observed in the WT Columbia (Figure 1). It should be noted that these mutants already display a constitutive Fe deficiency response and *IMA* expression under Fe-sufficient conditions could also be constitutively up-regulated. To confirm this possibility, we compared *IMA* expression under Fe-sufficient conditions between WT Columbia and the two mutants. Just as expected, we found that all the *IMA* genes were up-regulated in both the *frd3-3* and *opt3-2* mutants (Figure 2). These results would explain why both mutants present constitutively induced ferric reductase activity (Figure 3) and expression of the Fe acquisition genes *FRO2*, *IRT1* and *FIT* (García et al., 2013, 2018), since all of them are up-regulated by the IMA peptides (Grillet et al., 2018).

Previous results of our group showed that the application of FeSO_4_ to leaves of Fe-deficient WT Columbia and *frd3-3* plants greatly decreased their ferric reductase activity as well as the expression of the Fe acquisition genes *FRO2*, *IRT1* and *FIT* (Lucena et al., 2006; García et al., 2013, 2018). However, the foliar FeSO_4_ treatment did not significantly affect either ferric reductase activity or gene expression in Arabidopsis *opt3-2* plants (García et al., 2013), suggesting that the repressive “long-distance iron signal (LODIS)” can not move adequately from leaves to roots in the *opt3* mutant (García et al., 2018).

In this work, we studied the effect of the foliar application of FeSO_4_ on *IMA1, IMA2* and *IMA3* expression in the WT Columbia and in its LODIS-deficient mutants *frd3-3* and *opt3-2.* As occurred with the Fe acquisition genes (García et al., 2013), foliar application of Fe drastically inhibited *IMA* gene expression in WT and in *frd3-3* mutant plants, not restricted in the movement of LODIS from shoots to roots (Figure 4). However, it had no effect on *IMA* gene expression in *opt3-2* mutant plants, restricted in such a movement (Figure 4). These results clearly suggest that LODIS is involved in the regulation of *IMA1, IMA2* and *IMA3* expression.

### Ethylene involvement in the regulation of *IMA* gene expression

To investigate the possible relationship between ethylene and IMA peptides, both of them positive regulators of Fe deficiency responses, we carried out several assays in which Arabidopsis WT Columbia plants were subjected to ACC (ethylene precursor) or CoSO_4_ (Co, an inhibitor of ethylene synthesis) treatments (Lucena et al., 2006).

As shown in Figure 6, ACC treatment had a positive effect in the expression of the Fe acquisition genes *FRO2* and *IRT1*, at 6 and 24 h. However, in the case of *FIT*, the key TF that regulates *FRO2* and *IRT1*, the induction of its expression could only be observed at 24 h after ACC treatment (Figure 6).

*IMA1*, *IMA2* and *IMA3* expression experimented a fast induction after 6 h of ACC treatment (Figure 6). However, the positive effect of ACC treatment on *IMA* expression disappeared after 24 h (Figure 6).

To further verify the possible role of ethylene in the regulation of *IMA* genes, we studied the effect of Co treatment on their expression in Fe-deficient WT Columbia plants. We found that Co inhibited *IMA1*, *IMA2* and *IMA3* expression at all doses used: 50, 75 and 100 μM (Figure 5). Taken together, all these results clearly show that ethylene could participate in the regulation of *IMA1*, *IMA2* and *IMA3* genes.

### Occurrence of ethylene-responsive cis-acting elements in the promoters of *IMA* genes

The occurrence of ethylene-responsive cis-acting elements in the promoters of the *IMA* genes was investigated as additional evidence of their regulation by ethylene. The 8 bp sequence AWTTCAAA, is a well-known ethylene-responsive cis-acting element (ERELEE4 motif) that has been reported to mediate the ethylene regulation of genes for fruit ripening and leaf senescence (Montgomery et al., 1993; Itzhaki et al., 1994).

**Figure 7:**
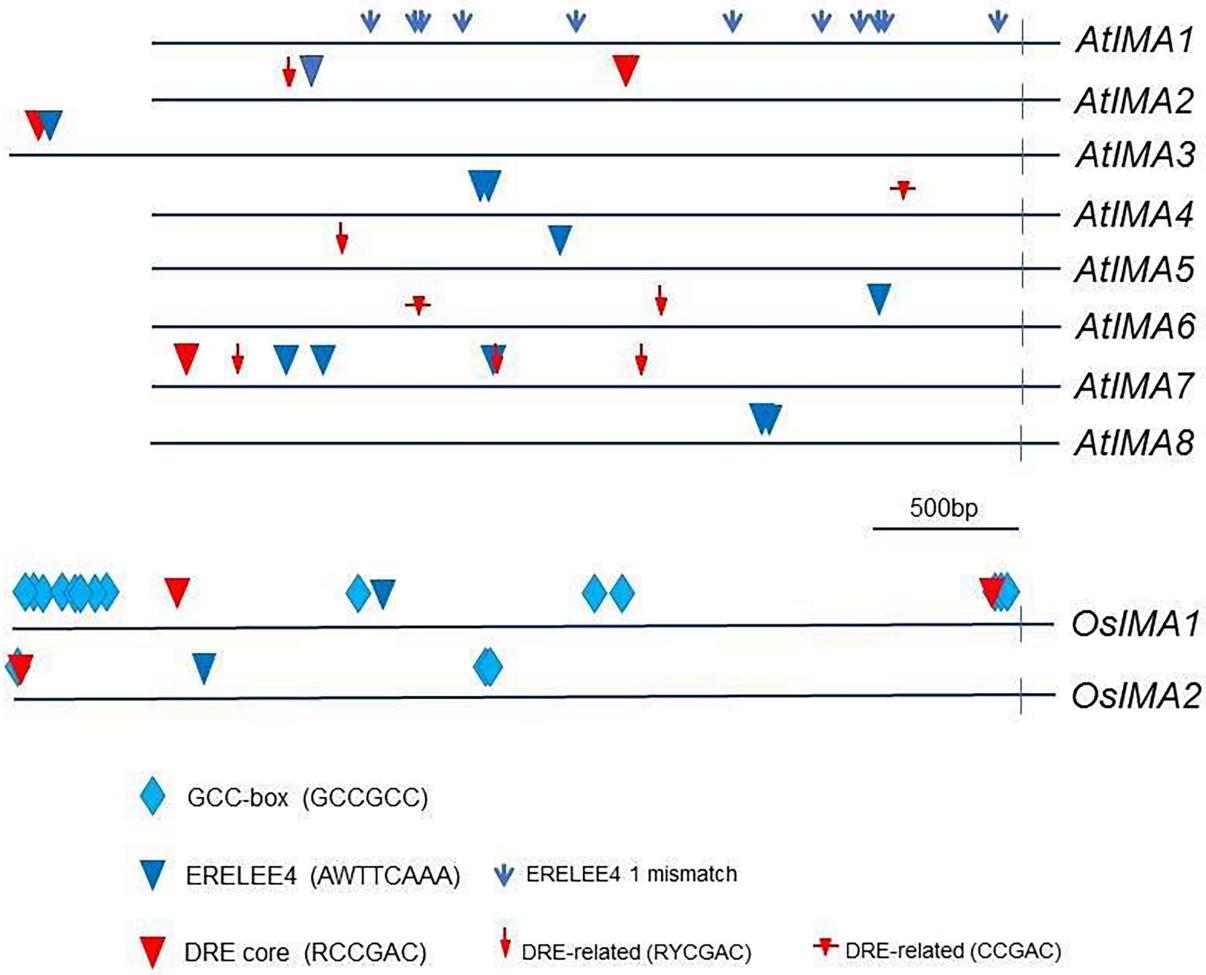
Location of ethylene-responsive motifs in the promoter regions of *IMA* genes. Ethylene-responsive motifs in the promoters of IMA genes were located by using the PLACE (Plant Cis-acting Regulatory DNA Elements) database. A stretch of 3,000 bp upstream of the transcription initiation site was examined in Arabidopsis (*AtIMA*) and in rice (*OsIMA*) sequences. For *AtIMA3* and *OsIMA1* and *OsIMA2*, the surveyed stretch was extended to 4,000 bp. The sequences of the promoter regions of the *IMA* genes were obtained from the EnsemblPlant and the TAIR database. *AtIMA1* (AT1G47400), *AtIMA2* (AT1G47395), *AtIMA3* (AT2G30766), *AtIMA4* (AT1G07367), *AtIMA5* (AT1G09505), *AtIMA6* (AT1G07373), *AtIMA7* (AT2G00920), *AtIMA8* (AT1G47401), *OsIMA1* (Os01g0647200), *OsIMA2* (Os07g0142100).

All eight Arabidopsis *IMA* genes, excepting *IMA1*, displayed at least one conserved ERELEE4 motif (Figure 7). Nevertheless, semi-conserved ERELEE4 motifs (one mismatch allowed) are present 11 times in the promoter of *IMA1* (Figure 7).

ERF1 is a transcription factor from the AP2/ERF family and is a fundamental part of the ethylene signaling pathway (Solano et al., 1998). The DRE core motif, RCCGAC, has been identified as a target sequence for ERF1 in Arabidopsis. By means of the DRE core motif, ethylene regulates gene expression in response to drought, salt, and thermal stresses (Cheng et al., 2013).

Cis-acting elements RYCGAC and CCGAC are close related to the DRE core (RCCGAC) and have also been identified as target sequences for AP2/ERF transcription factors (Hao et al., 2002; Sakuma et al., 2002; Xue, 2003; Lata and Prasad, 2011). Those DRE core-like sequences control gene expression in response to drought and cold in barley (Xue, 2003) and Arabidopsis (Sakuma et al., 2002; Lata and Prasad, 2011).

The DRE core motif and related elements were detected in all Arabidopsis *IMA* genes except in *IMA1* and *IMA8* (Figure 7). Moreover, six out of eight Arabidopsis *IMA* genes, including *IMA2* and *IMA3*, contain both types of ethylene-responsive elements, ERELEE4 and DRE (Figure 7).

*IMA* orthologues in rice, *OsIMA1* and *OsIMA2*, have been recently characterized by Kobayashi et al. (2021). Both genes are strongly induced under Fe deficiency and play key roles in enhancing the Fe deficiency responses. However, whether ethylene can regulate *OsIMA1* or *OsIMA2* expression or not, is yet unknown.

We have extended our identification of ethylene responsive cis-acting elements to *OsIMA1* and *OsIMA2*. The promoters of each of them contain both ERELEE4 and DRE core motifs (Figure 7). Moreover, both *OsIMA1* and *OsIMA2* also display multiple copies of another well-characterized ethylene responsive cis-acting element called the GCC box (GCCGCC; Hao et al., 1998). This motif is especially abundant in *OsIMA1*, which contain 19 copies (Figure 7).

## Discussion

IMA peptides were found few years ago in Arabidopsis and described as key positive regulators of Fe deficiency responses in Strategy I plants (Grillet et al., 2018; Hirayama et al., 2018). This finding has been further supported by other authors (Gautam et al., 2021; Li et al., 2021; Lichtblau et al., 2022), representing a great advance in the knowledge about the complex regulatory system of the Fe deficiency responses. Recently, the IMA peptides have also been described in rice (Kobayashi et al., 2021; Peng et al., 2022).

Previously to the discovery of IMA peptides, different hormones and signaling substances, such as ethylene, auxin, GSH and NO, have also been involved in the induction of Fe deficiency responses through the activation of FIT and other TFs (Lucena et al., 2006, 2015; Graziano and Lamattina, 2007; Waters et al., 2007; Chen et al., 2010; García et al., 2010, 2011, 2018; Lingam et al., 2011; Meiser et al., 2011; Romera et al., 2011, 2017; Koen et al., 2012; Yang et al., 2014; Shanmugam et al., 2015; Li and Lan, 2017). Besides these activating signals, inhibitory signals, like cytokinins and Fe itself, have also been described as necessary to switch off the responses once sufficient Fe has been acquired (Lucena et al., 2006; Séguéla et al., 2008; García et al., 2013, 2018). In relation to Fe itself, the repressing signal has been associated with some Fe compound circulating from shoots to roots through the phloem and related to the OPT3 transporter (García et al., 2013, 2018; Mendoza-Cózatl et al., 2014; Zhai et al., 2014). Probably, this Fe repressing signal is either a substrate of the OPT3 transporter or is derived from it (García et al., 2018). This **lo**ng-**d**istance inhibitory **i**ron **s**ignal has been named “LODIS” and integrated with activating signals, such as ethylene, in a recent model about regulation of Fe deficiency responses (García et al., 2018). After the discovery of the IMA peptides, our group have been working in trying to fit them in the previous model (García et al., 2013, 2018). To this end, it is necessary to decipher the possible relationship between LODIS and IMA peptides, and between IMA peptides and ethylene.

To look further in the LODIS-IMA relationship, we carried out several experiments with the Arabidopsis WT Columbia and its LODIS defective mutants *opt3-2* and *frd3-3* (see Materials and Methods). First, we compared the expression profiles of *IMA1, IMA2* and *IMA3* in roots of WT *Columbia* and its two LODIS defective mutants subjected to Fe-deficient conditions. The results obtained showed a great increment of the expression of *IMA* genes in WT Columbia after 12 h of Fe deficiency, reaching their maximum expression level at 24 h, up to 30–40 fold (Figure 1). This increment was associated with an enhanced ferric reductase activity, one of the Fe deficiency responses and a target of IMA peptides (Grillet et al., 2018), at 48 h (Figure 3). On the other side, Fe deficiency did not induce either a great increase of the expression of *IMA* genes (only about 3–4 fold), nor of ferric reductase activity, in the *frd3-3* and *opt3-2* mutants (Figures 1, 3). These results could be explained by taking into account that the *frd3-3* and *opt3-2* mutants present constitutive expression of Fe deficiency responses (García et al., 2013, 2018), like the ferric reductase activity (Figure 3), and also constitutive expression of the *IMA* genes (Figure 2). In supporting these latter results, Chia et al. (2021) have also found up-regulation of *IMA2* expression in Fe-sufficient roots of *opt3-2* plants. Taking into account that *frd3-3* and *opt3-2* are LODIS defective mutants (see Material and Methods), the results depicted above suggest that LODIS is involved in the regulation of *IMA* expression. To further confirm the role of LODIS on *IMA* regulation, we performed several experiments in which WT Columbia, *frd3-3* and *opt3-2* plants were subjected to foliar Fe treatment, to provide leaves with enough LODIS to export to the roots. The results obtained showed that the induction of *IMA* expression was drastically abolished by the foliar Fe treatment in WT Columbia and in *frd3-3* mutant plants grown under Fe-deficient conditions (Figure 4). This further supports an inhibition of *IMA* expression by LODIS. However, the foliar application of Fe had no effect in the mutant *opt3-2* (Figure 4), just as occurred with Fe acquisition genes (*FRO2, IRT1*, and *FIT*) in previous works (García et al., 2013, 2018). It should be noted that the *opt3-2* mutation impairs LODIS exportation from leaves to roots while the *frd3-3* mutation does not (García et al., 2018). All these results clearly suggest that LODIS is involved in the negative regulation of *IMA* expression.

The second objective of this work was to determine the possible relationship between two activating signals of the Fe deficiency responses: IMA peptides and ethylene. Previous results of our group showed that ACC (ethylene precursor) requires low Fe concentration in the medium to activate Fe deficiency responses (Lucena et al., 2006). In this work, we have found that ACC application to WT Columbia plants grown on low Fe conditions (Fe 10 μM) had a positive effect on the Fe acquisition genes (*FRO2* and *IRT1*) at both times studied (6 and 24 h; Figure 6). However, a significant induction of *FIT* expression could only be observed at 24 h after ACC treatment (Figure 6). The fact that *FIT* expression increase takes place later than that of their target genes *FRO2* and *IRT1* may seem puzzling. Nevertheless, Balparda et al. (2020) have recently proposed that *FRO2* and *IRT1* expression could be also directly controlled by the ERF1 TF (an essential part of the ethylene signaling) in a FIT independent way.

The expression of the *IMA* genes studied in this work increased in a significant manner 6 h after ACC treatment (Figure 6). This positive effect of ACC on the *IMA* expression disappeared quickly, in such a way that 24 h after ACC treatment no effect was observed (Figure 6). These results clearly suggest that ethylene positively regulates *IMA* expression, at least transitorily. To corroborate this, we further studied the expression of *IMA* genes in WT Columbia Fe-deficient plants subjected to an ethylene synthesis inhibitor, like cobalt (CoSO_4;_ Lucena et al., 2006). The results showed that Co, at all doses used, inhibits the induction *IMA* expression in Fe-deficient plants (Figure 5), thus corroborating a possible role for ethylene in the up-regulation of *IMA* expression.

Finally, as and additional proof of ethylene involvement on *IMA* regulation, we investigated the occurrence of ethylene-responsive cis-acting elements in the promoters of *IMA* genes in both Arabidopsis and rice. Interestingly, the eight Arabidopsis *IMA* genes, excepting *IMA1*, displayed at least one conserved ERELEE4 motif (Figure 7). On the other side, in the promoter of *IMA1,* semi-conserved ERELEE4 motifs (only one mismatch allowed) are present 11 times (Figure 7). The ERELEE4 motif has also been found in the promoters of 14 Fe-related genes regulated by ethylene (García et al., 2010). Some of them are essential genes for the regulation of Fe deficiency responses (*FIT, bHLH38*), Fe acquisition (*IRT1*) and Fe distribution (*FRD3, NAS1* and *NAS2*). The DRE core motif, RCCGAC, is another ethylene-responsive cis-acting element (Cheng et al., 2013). DRE core or related motifs occur in all Arabidopsis *IMA* genes but *IMA1* and *IMA8* (Figure 7). Moreover, six out of eight Arabidopsis *IMA* genes, including the highly expressed *IMA2* and *IMA3* (Grillet et al., 2018), contain both types of ethylene-responsive cis-acting elements, ERELEE4 and DRE core (Figure 7). Taken together, all these results clearly support a role for ethylene in the regulation of *IMA* expression and provide a way to know how ethylene could regulate them. It is interesting to consider that, since *IMA* genes seem to play redundant roles in triggering Fe deficiency responses (Grillet et al., 2018), the regulation by ethylene of just one of them, especially the highly expressed *IMA1*, *IMA*2 or *IMA*3, will be enough to establish a control by ethylene of the *IMA* gene function. Ethylene-responsive cis-acting elements are also found in the promoters of rice *IMA* genes (Figure 7), which further supports the role of ethylene in the regulation of IMA peptides.

**Figure 8:**
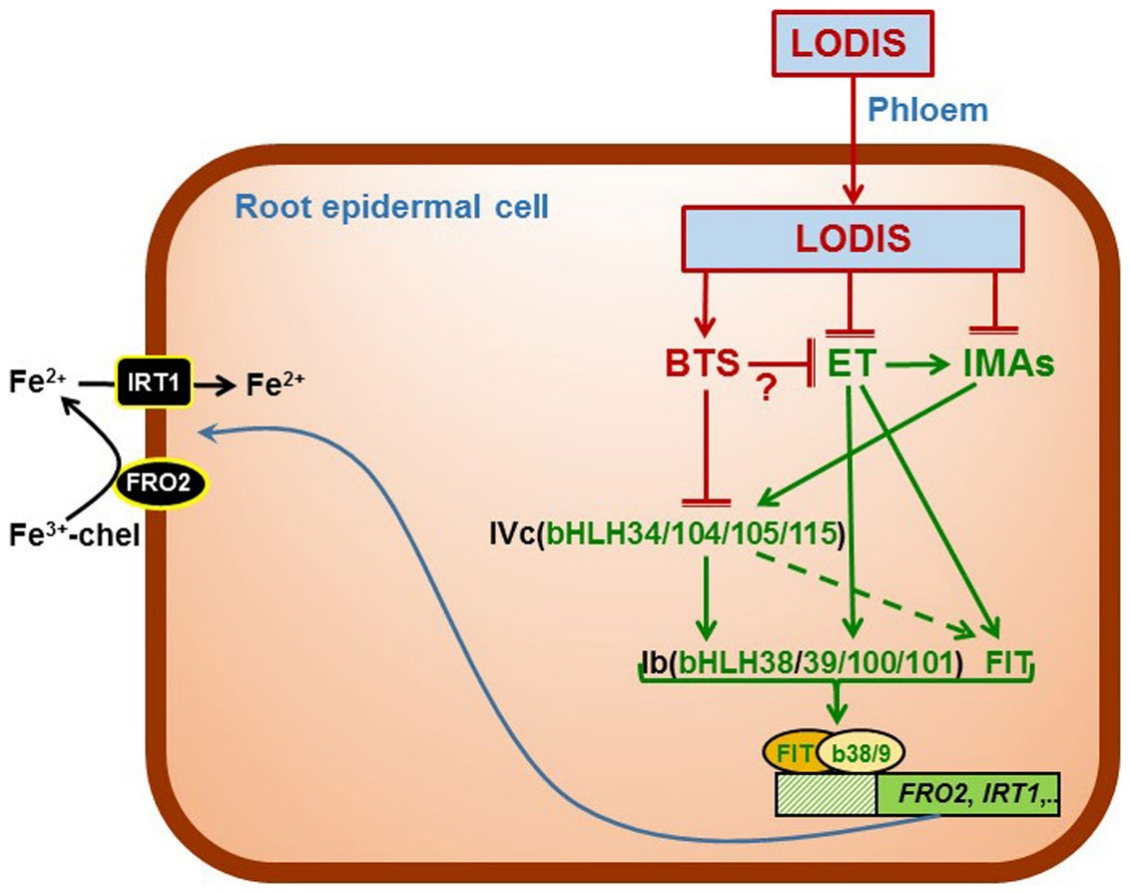
Working model to explain the role of LODIS, IMA peptides and ethylene on the regulation of Fe acquisition genes. Once in shoots, some Fe (either as free ions or in chelated form) can enter the phloem through the OPT3 transporter and move back to roots bound to a chelating agent (forming LODIS). In roots, LODIS could negatively affect *IMA* expression and ethylene synthesis and signaling, either directly or through BTS. Ethylene can affect *IMA* expression as well as the expression of *FIT* and *Ib-bHLH* expression. Both IMAs and BTS interact to regulate IVc-bHLH activity, which influence Ib-bHLHs and FIT and, consequently, Fe acquisition genes. ET (ethylene), IMAs (IMA peptides). (→: promotion; ⊥: inhibition).

In conclusion, the results obtained in this work suggest that LODIS could act upstream of IMA peptides and ethylene as depicted in the Working Model of Figure 8. According to this model, modification of a previous one presented by García et al. (2018), once Fe gets to shoots, LODIS is formed and moves to roots through the phloem. In roots, LODIS could negatively affect *IMA* expression and ethylene synthesis and signaling, either directly or through BTS. In a previous work, it was found that LODIS can affect ethylene production in roots since several Arabidopsis LODIS defective mutants, such as *op3-2*, *frd3-3* and *nas4x-1*, present higher ACC (ethylene precursor) accumulation in Fe-sufficient roots than the WT (García et al., 2018). Since BTS is a putative sensor of Fe (probably of LODIS), then it is also possible that BTS could influence ethylene production. In supporting this view, it should be noted that the *SAM1* gene, encoding a SAM synthetase (involved in ethylene synthesis), is up-regulated in the *bts-3* mutant (Hindt et al., 2017). In addition, the phenotype of the *bts-3* mutant could also be related to ethylene since it presents shorter roots and small shoots (Hindt et al., 2017), as plants treated with ethylene. Ethylene itself can also affect *IMA* expression as well as the expression of *FIT* and that of the *bHLHs* of the Ib subgroup (García et al., 2010; Lucena et al., 2015). Both IMAs and BTS interact to regulate IVc-bHLHs activity, which activate Ib-bHLHs and FIT, and consequently Fe acquisition genes (Li et al., 2021; Lichtblau et al., 2022).

This work has been focused in the study of the possible relationship between IMA peptides and other known signals implicated in the regulation of Fe deficiency responses, like LODIS and ethylene. Evidence for a role of LODIS and ethylene in the regulation of *IMA1, IMA2* and *IMA3* expression in Arabidopsis is presented. Nevertheless, there is still a long way to go. We have studied the effect of LODIS and ethylene on *IMA1, IMA2*, and *IMA3* expression, but further studies are necessary to clarify other possibilities, like this one: is ethylene influenced by IMA peptides? Another open question to be solved is the possible relationship between NO and IMA peptides. Ethylene and NO act in conjunction to regulate Fe deficiency responses (García et al., 2011), hence it is possible that NO could also participate in *IMA* regulation.

## Data availability statement

The datasets presented in this study can be found in online repositories. The names of the repository/repositories and accession number(s) can be found in the article.

## Author contributions

MG and FR designed the experiments after discussions with RP-V. MG, MA, and CL conducted the laboratory work. MA drew the figures and did the statistical data analyses. RP-V did the search for DNA motifs in IMA genes. MG, FR, and RP-V wrote the manuscript which was improved by the other authors. All authors contributed to the article and approved the submitted version.

## Funding

This work was supported by the European Regional Development Fund from the European Union, the ‘Ministerio de Economía y Competitividad’ (Project RTI2018-097935-B-I00), and the ‘Junta de Andalucía’ (Research Groups AGR115 and BIO159).

## Conflict of interest

The authors declare that the research was conducted in the absence of any commercial or financial relationships that could be construed as a potential conflict of interest.

## Publisher’s note

All claims expressed in this article are solely those of the authors and do not necessarily represent those of their affiliated organizations, or those of the publisher, the editors and the reviewers. Any product that may be evaluated in this article, or claim that may be made by its manufacturer, is not guaranteed or endorsed by the publisher.

## Footnotes

^1^ https://www.dna.affrc.go.jp/PLACE/?action=newplace

^2^ https://plants.ensembl.org/index.html

^3^ https://www.arabidopsis.org/
